# The complete chloroplast genome of *Epimedium enshiense* B. L. Guo et Hsiao (Berberidaceae)

**DOI:** 10.1080/23802359.2020.1730269

**Published:** 2020-05-12

**Authors:** Xiaoming Li, Tianrong Xu, Yu Yao, Qianru Yang, Chaoqun Xu, Fengmei Suo, Cheng Zhang, Guoan Shen, Baolin Guo, Xiang Liu, Shenghua Wei

**Affiliations:** a School of Pharmacy, Guizhou University of Traditional Chinese Medicine, Guiyang, China; b Institute of Medicinal Plant Development, Chinese Academy of Medical Science, Peking Union Medical College, Beijing, China; c College of Pharmacy, Tianjin University of Traditional Chinese Medicine, Tianjin, China; d Chongqing Key Laboratory of Traditional Chinese Medicine Resource, Chongqing Academy of Chinese Materia Medica, Chongqing, China

**Keywords:** Chloroplast genome, *Epimedium enshiense*, Berberidaceae

## Abstract

The genus of *Epimedium* belongs to Berberidaceae family, which is famous for their medicinal and ornamental value. In recent years, *Epimedium* has attracted increasing attention due to their medicinal and nutritive value. In this research, we reported the complete chloroplast (cp) genome of *Epimedium enshiense.* The complete chloroplast of this species is 157,076 bp in length, including a pair of invert repeat regions (IRS) (25,833 bp) that is divided by a large single copy area (LSC) (88,340 bp) and a small single copy area (SSC) (17,070 bp). The circular chloroplast genome of *E*. *enshiense* contains 112 unique genes, composing of 78 protein-coding genes, 30 tRNA, and four rRNA genes. Phylogenetic analysis indicates that *E*. *enshiense* has a closer relationship with *E. dolichostmon*.

*Epimedium* is a genus of herbaceous plants in the Berberidaceae family, which was used as traditional medicinal plants for more than 2000 years in China (Zhao et al. 2012). *Epimedium enshiense* B. L. Guo et Hsiao is a rare species with yellow spider-like flowers, which only inhabits in the Enshi County, Hubei Province, China (Guo and Xiao 1993). In this study, we reported the complete chloroplast sequence of *E. enshiense*, and analyzed the relationship between *E. enshiense* and other *Epimedium* species by phylogenetic analysis.

The sample of *E*. *enshiense* was collected from the Enshi County, Hubei province, China (E108°42, N28°26′). The voucher specimen (Guo0464) was deposited at the Herbarium of the Institute of Medicinal Plant (IMPLAD), Beijing, China. Total genomic DNA was extracted from the fresh leaves of *E*. *enshiense* with the modified CTAB method (Doyle and Doyle 1987). DNA library was sequenced, and 150 bp paired-end reads were generated on an Illumina Novaseq PE150 platform. The clean reads were assembled by using the program GetOrganelle v1.5 (Jin et al. 2018) with the reference chloroplast genome of *E. acuminatum* (GenBank accession number: KU522469). The chloroplast genome annotation was conducted through the online program CPGAVAS2 (Shi et al. 2019) and GeSeq (Tillich et al. 2017). The annotated chloroplast genomic sequence has been registered in GenBank with an accession number (MN937557).

The complete chloroplast genome of *E*. *enshiense* is 157,076 bp in length, and has a typical quadripartite construction, which contains two inverted repeat regions (IRa and IRb) of 25,833 bp that is insulated by a large single-copy (LSC, 88,340 bp) and a small single-copy (SSC, 17,070 bp). The total GC content of complete chloroplast genome, LSC, SSC, IR regions is 38.81%, 37.43%, 32.74% and 43.19%, respectively. The complete chloroplast genome of *E*. *enshiense*is contains 112 unique genes, including 78 protein-coding genes, 30 tRNA genes and four rRNA genes. Most of these genes are single-copy genes. However, four protein-coding genes (*rps*7, *ndhB*, *ycf*2, and *rp1*23), seven tRNAs (*trnI*-*CAU*, *trnL*-*CAA*, *trnV*-*GAC*, *trnI*-*GAU*, *trnA*-*UGC*, *trnR*-*ACG*, and *trnN*-*GUU*), and four rRNAs (*rrn*16, *rrn*23, *rrn*4.5, *and rrn*5) are duplicated in the IR regions. One tRNA gene (t*rnQ*-*UUG*) repeated in the LSC regions. In these genes, 15 genes (six tRNA genes and nine protein-coding genes) contain one intron, whereas three genes (*ycf*3, *clpP*, and *rps*12) contain double introns. The *rps*12 gene is trans-spliced, with the 5′ end located in the LSC region, and the 3′ end duplicated in the IR region.

To confirm the phylogenetic position of *E. enshiense*, the complete chloroplast genomes of 19 other plant species were downloaded from the NCBI GenBank database. The sequences were aligned using MAFFT v7 (Katoh et al. 2017), and then the maximum likelihood tree (Figure 1) was constructed using raxmlGUI1.5b (v8.2.10) (Silvestro and Michalak 2012). Phylogenetic analysis shows that *E. enshiense* is closely related to *E. dolichostemon*. The published *E. enshiense* chloroplast genome provides useful information for phylogenetic and evolutionary studies in Berberidaceae.

**Figure 1.**
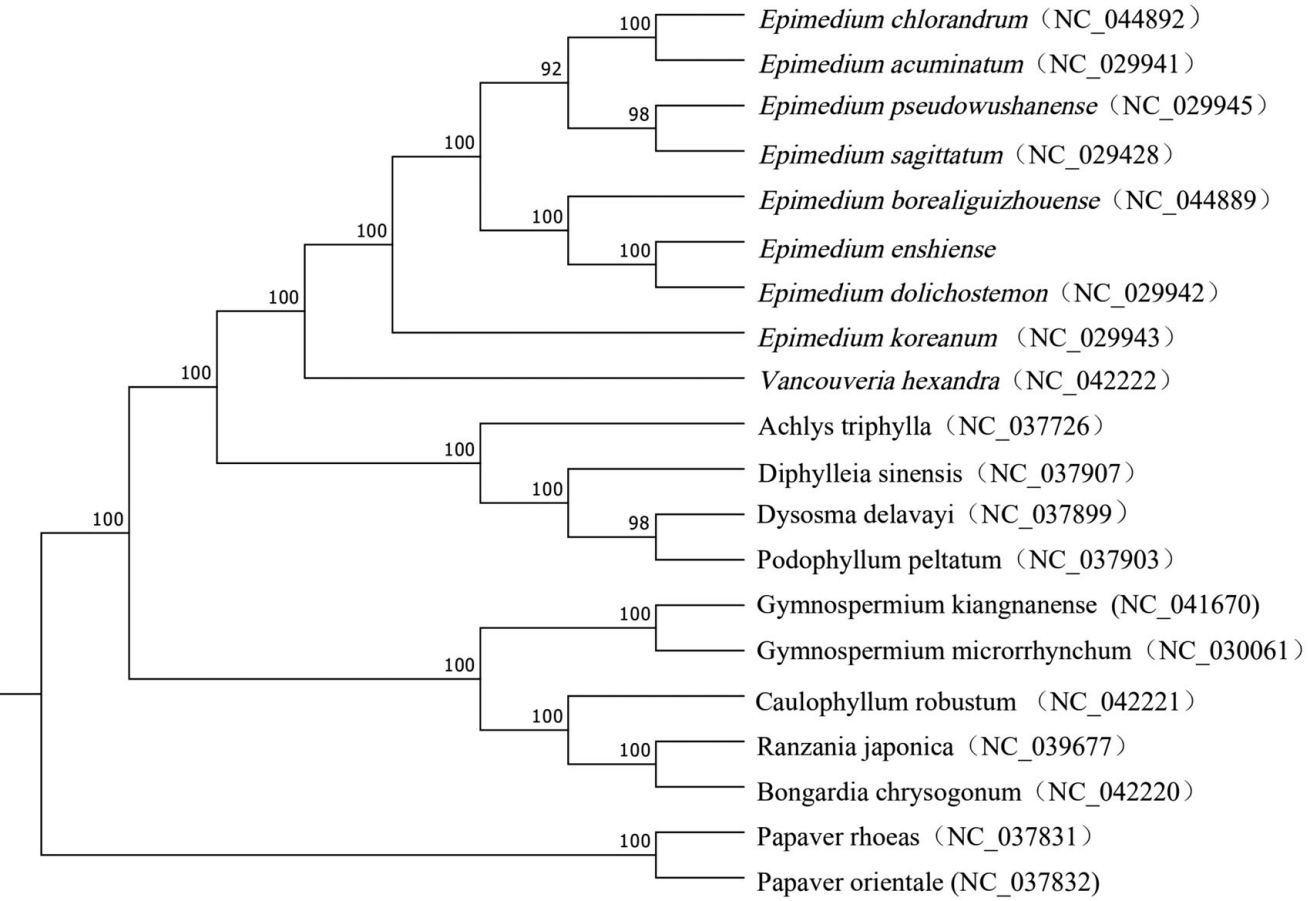
The Maximum likelihood (ML) phylogenetic tree based on complete chloroplast genomes of 20 species, with *Papaver rhoeas and Papaver orientale* as outgroup. The numbers above the lines represent ML bootstrap values.
